# Eye-tracking biomarkers for glaucoma based on saccadic reaction time: a controlled clinical study

**DOI:** 10.3389/fopht.2025.1636911

**Published:** 2025-10-16

**Authors:** Alexander Sverstad, Bjørn André Helland-Hansen, Olav Kristianslund, Miriam Kolko, Stig Einride Larsen, Goran Petrovski

**Affiliations:** 1 Centre for Eye Research and Innovative Diagnostics, Oslo University Hospital (OUS), Ullevål, Oslo, Norway; 2 Department of Ophthalmology, Vestfold Hospital Trust, Tønsberg, Norway; 3 Department of Ophthalmology, Oslo University Hospital and Institute of Clinical Medicine, University of Oslo, Oslo University Hospital (OUS), Ullevål, Oslo, Norway; 4 Department of Drug Design and Pharmacology, University of Copenhagen, Copenhagen, Denmark; 5 Department of Ophthalmology, Rigshospitalet, Glostrup, Denmark; 6 Meddoc Research, Skjetten, Norway

**Keywords:** glaucoma, visual field test, eye movement perimetry, saccadic reaction time, reliability, biomarkers, stability index, agreement index

## Abstract

**Purpose:**

Evaluate the validity and reliability of saccadic reaction time (SRT)-based variables obtained using the novel eye-tracking device Bulbicam (BCAM) in differentiating early-to-moderate glaucoma (GLA) from healthy controls (HCs) and to identify potential biomarkers for GLA.

**Methods:**

A controlled clinical study was conducted, involving 18 GLA-patients, and 18 age-matched HCs. Participants underwent BCAM’s visual field (VF) test, which measures SRT at 58 symmetrically arranged locations with 6° spacing. Variables were analysed for group differences, within- and between-patient repeatability, and stability. To evaluate their potential as biomarkers, VF locations were aggregated into clusters, quadrants, hemifields, and whole VF analyses.

**Results:**

Significant SRT differences (p ≤ 0.05) were observed between GLA and HC in 44 of 58 locations in the worst eye and 42 of 58 in the best eye. Eight out of ten clusters met the criteria for BCAM biomarkers having significant group differences, sufficient within- and between-patient repeatability, and adequate stability. All quadrants demonstrated excellent stability and repeatability thereby qualifying as biomarkers. Hemifield SRTs were reliable, however, the absolute difference between hemifields showed poor within-participant repeatability. The mean and standard deviation of SRT for the whole VF were identified as significant biomarkers with excellent stability.

**Conclusions:**

The majority of SRT variables are capable of differentiate glaucomatous eyes from HC while maintaining sufficient reliability and stability for clinical application. 19 of 22 BCAM VF test variables were found to be potential GLA-biomarkers.

**Clinical Trial Registration:**

https://clinicaltrials.gov/, identifier NCT05449041.

## Introduction

Glaucoma (GLA) is a debilitating disease characterised by the loss of retinal ganglion cells (RGCs) and their axons, which may ultimately lead to blindness (1–3). The asymptomatic nature and time-consuming assessment of this disease pose significant challenges for timely diagnosis and management. Despite advancements in technology such as optical coherence tomography (OCT) and standard automated perimetry (SAP), uncertainty in diagnosing and monitoring disease progression persists (4). SAP, the most widely used functional test (5), fails to detect damage until roughly 30% of retinal ganglion cells are lost (6–8), and it’s variability further complicates clinical decision-making (9–11). These limitations highlight the need for innovative diagnostic strategies capable of detecting GLA earlier and with greater reliability.

Eye-tracking technology has been widely used to study ocular motor control and to investigate the impact of neurological and ophthalmological diseases on eye movement behaviour (12, 13). In GLA, the influence on saccadic reaction time (SRT) has been well documented. Kanjee et al. were the first to explore this in 2012 (14), demonstrating prolonged saccadic latencies across all disease stages, and numerous subsequent studies have confirmed these findings (15–20), including analyses in specific GLA subgroups (21). Compared to SAP, SRT-based perimetry has showed comparable clinical applicability (17). A key distinction between SAP and SRT-based perimetry lies in the mode of patient interaction. SAP relies on subjective button-press responses and sustained, stable fixation, which are vulnerable to inattention, fatigue and response bias, contributing to variability. In contrast, SRT-based perimetry exploits reflexive eye movements that are rapid, and closely tied to visual processing, thereby reducing cognitive load and improving engagement. While SRT is known to be significantly affected by factors such as age and stimulus characteristics, it appears to show small variation with respect to ethnicity, sex, or the presence of cataract (22–24). Importantly, some studies have highlighted the potential of SRT-based perimetry as a promising method for early GLA detection, with evidence showing its ability to detect decreased VF responsiveness in regions that appear normal on SAP (25–27).

Despite this encouraging evidence, SRT-based eye movement perimetry (EMP) has yet to be adopted in routine clinical practice. Barriers include the lack of standardisation, and formal validation across platforms, as well as practical constraints of some existing systems, which can involve more extensive setup procedures and longer testing times. Furthermore, most published studies have prioritized demonstrating differences between GLA and HCs, but relatively little attention has been paid to measurement reliability and stability. To date, only one study, conducted by Pel et al. in 2013, has specifically addressed the validity and repeatability of SRT-based perimetry, limited to a healthy population, demonstrating low variability in SRT across three repeated measurements (28).

Over time, advancements in eye-tracking technology have led to a range of solutions, from desktop display systems to compact, portable devices resembling virtual reality (VR) goggles. One such innovation is a device called Bulbicam (BCAM), developed by Bulbitech (Trondheim, Norway). Designed as a point-of-care tool, BCAM integrates a high-precision eye-tracking system with dual displays in a VR-goggle-like format, facilitating in-depth assessment of visual and neurological function through a diverse set of tests. BCAM features a user-friendly interface, allowing for rapid test administration, intuitive result interpretation, and flexible adaptation to various clinical and research settings. Of particular relevance for GLA assessment is BCAM’s visual field (VF) test, a perimetric test based on SRTs measured across a symmetric grid of 58 locations with 6°spacing. While perimetry exists in other VR-goggle-like platforms, they are typically based on light sensitivity where the responses are given by eye movements or button presses. BCAM is among the first VR-goggle-like platform to incorporate VF responsiveness in the form of SRT-based perimetry.

As with any new diagnostic tool, the value of SRT-based perimetry depends not only on its ability to distinguish patients from HCs but also on the extent to which its measurements are affected by error. Establishing the validity, reliability, and stability of test variables is essential before they can be considered for clinical adoption (29). Reliability reflects the consistency of measurements and requires repeatability within- and between subjects. In practice, within-subject repeatability is often assessed using Bland-Altman plots, whereas between-subject repeatability is commonly expressed using the intraclass correlation coefficient (ICC). Stability describes the consistency of measurements over multiple time points and can uncover training effects or temporal variability that may influence longitudinal monitoring. Together, these properties determine whether a variable has the quality required to function as a clinically meaningful biomarker or not.

While the literature has consistently demonstrated that SRTs are prolonged in GLA, to our knowledge, no study to date has specifically focused on the reliability and stability of these metrics in a GLA population, nor formally assessed their potential as biomarkers. This gap may, in part, reflect challenges in standardising the criteria required to qualify biomarkers (30). Within our framework, a variable qualifies as a biomarker only if it demonstrates sufficient validity and reliability for a clearly specified diagnostic purpose within a defined test procedure.

The aim of this study was to assess BCAM’s VF test in terms of validity, reliability, and to explore the potential of 22 predefined variables as biomarkers for GLA.

## Materials and methods

### Materials

The study sample comprised 18 patients diagnosed with early-to-moderate open-angle GLA, and 18 age-matched healthy controls (HCs) of both genders, at least 18 years of age, and without any other eye disease or other known serious systemic disease. Three screened candidates were excluded prior to enrolment due to age-related macular degeneration, Parkinson’s disease, and epiretinal fibrosis and are not counted among the 18 included patients. Out of these, six eyes were excluded. One due to previous retinal vein occlusion, and the rest due to no detectable GLA changes or advanced GLA. GLA severity was defined by mean deviation (MD) from SAP. Early GLA was defined as MD ≤ 6 dB, and moderate as MD > 6 dB and ≤ 12 dB (5). Excluded participants included those with best corrected visual acuity (BCVA) worse than 1.0 logMAR in either eye, inability to perform eye movements, abnormal visible part of the eye, pupils not able to respond normally to dilation or contraction (e.g., due to damaged nerves or mechanical damage of the pupil). IOP was not used as an inclusion criterion, as participants were already diagnosed and under routine follow-up. Participants were consecutively recruited from the Department of Ophthalmology, Oslo University Hospital Ullevål and Vestfold Hospital Trust. For each participating patient, an age-matched HC without neurological or ophthalmological disease was included. To maintain statistical power, and allow for independent analysis, each participant’s eyes was categorised as ‘best’ or ‘worst’ based on MD from SAP, with BCVA used as a secondary criterion if MD was equal in both eyes. In the six cases where only one eye was included, the same eye was used in both best and worst eye analyses. This was also applied to the HCs group to better balance the analyses. In total, 18 eyes were classified as mild (out of which 3 were preperimetric) and 12 as moderate GLA. In the best eye analysis, 10 were mild and 8 moderate, while in the worst eye analysis, 9 were mild and 9 moderate. Demographic and clinical characteristics of the study participants can be found in **Table 1**.

**Table 1:** Demographic and clinical characteristics of study participants.

Factor/ variables		Glaucoma patients (GLA)	Heathy controls (HC)
Demographic factors	Sex (F/M)	10/8	13/5
	Age (years)	71.7 (58.9 - 84.5)	71.5 (58.9 - 83.7)
	Disease duration (years)	7.0 (0.5 - 20.9)	–
Clinical characteristics	MD	5.7 (-1.3 – 10.6)	2.0 (-0.7 – 5.6)
	IOP (mmHg)	13.9 (9.5 – 22.0)	13.6 (7.0 – 22.0)
	BCVA (logMAR)	0.0 (-0.2 - 0.2)	0.0 (-0.4 – 0.2)

### Ethics

All participants gave written informed consent. The study was approved by the institution data protection officer of Oslo University Hospital and Vestfold Hospital Trust. The study was considered by the Regional Ethics committee to be outside their mandate. The study adhered to the tenets of the Declaration of Helsinki and is registered with ClinicalTrials.gov (NCT05449041).

### Methods

This study was designed as an open, non-randomized, controlled clinical study.

#### Equipment

Data collection was performed using the BCAM device, which employs video-oculography technology, utilising both dark pupil/bright pupil tracking and corneal reflex techniques at a frequency of 400 frames per second (fps) to capture precise gaze direction data. The device features two liquid crystal displays and an infrared eye-tracking camera, enabling presentation of stimuli to one or both eyes and tracking accordingly, based on the test chosen.

#### Clinical procedure

Participants were seated comfortably and fitted with a mask designed to maintain the optimal distance between the eyes and the displays, while also blocking external light contamination. To further minimise light contamination, BCAM examinations were performed in a dimly lit room. Background noise was kept to a minimum. The mask was magnetically secured to the BCAM, which was positioned on a desk stand and adjusted to ensure participants could comfortably maintain a stable head position. Each participant’s interpupillary distance (IPD) and refraction for distance was entered into the BulbiHub software, which automatically calculated the appropriate refraction for the BCAM glasses.

**Figure 1:**
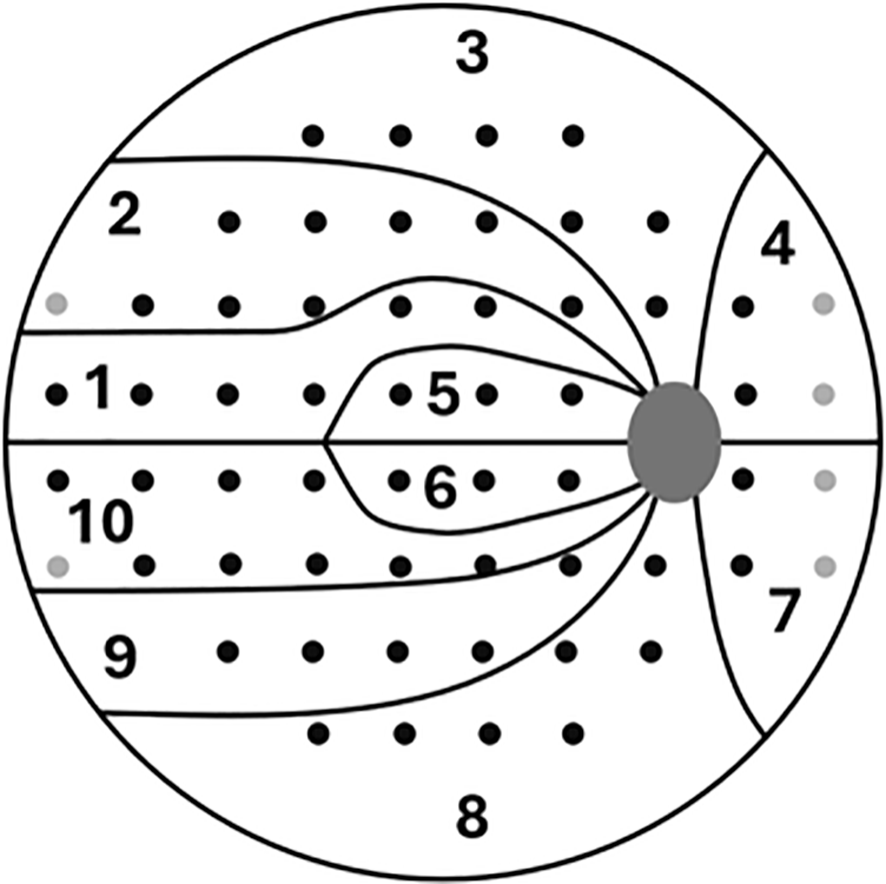
The BCAM VF test pattern with clusters. The grey points mark the difference from the 24–2 test pattern.

Participants completed a white-on-white, eye movement-based perimetry test, designated as the “VF test”. This test employs a grid pattern similar to the commonly used 24–2 layout with 6° spacing. In contrast to the 24–2 layout, it includes two additional peripheral nasal locations and four additional peripheral temporal locations, making a total of 60 test locations. For the analysis, locations at 15^0^ temporal, ± 3^0^ vertically were excluded as these locations correspond to the blind spot in most individuals (31) (**Figure 1**).

The stimulus was presented using the overlap paradigm, with a size of 0.43° (equivalent to Goldman size III) and flicker of 10 Hz, against a background luminance of 10 cd/m^2^. For each test location, the stimulus brightness logarithmically increased from 10cd/m^2^ to a peak of 262 cd/m^2^ over 3 seconds. A green circle, with a size of 0.66^0^, served as a fixation target; participants were instructed to maintain their gaze on this target until detecting the flickering white stimulus. Upon noticing the stimulus, they were instructed to immediately shift their gaze and fixate on it.

The SRT was measured as the time between the first frame displaying the stimulus, to the first frame in which the participants’ gaze deviated outside the fixation target. For an SRT to be accepted, the participant’s fixation had to remain within the green circle for a minimum of 120 frames and subsequently on the stimulus for 25 frames. If this criterion was not met, another trial was initiated.

BCAM assessments were completed over 1–3 days depending on participant convenience. Each BCAM VF test took approximately 2 minutes per eye, and participants were encouraged to take breaks as needed. GLA patients completed a total of six test repetitions, while HC completed two. Calibration was done automatically by the device’s software. All recordings were done by the same operator (AS). Participants received standardised instructions in Norwegian. To minimize the learning effect, every participant completed a minimum of two practice tests before formal data collection.

The variables in this study were SRTs measured at 58 predefined VF locations with the Bulbicam test (**Figure 1**). From these, we derived aggregated measures to better evaluate regional susceptibility to glaucomatous damage, enhance the clinical interpretability of spatial patterns in functional loss, and assess how reliability changes with different levels of spatial aggregation.

• Cluster 1 to 10 (**Figure 1**): Groups of anatomically and functionally related VF points, arranged similar to that used in the EyeSuite software (Haag-Streit Inc., Köniz, Switzerland) (32). These clusters approximate the distribution of RNFL bundles, an established approach in glaucoma evaluation.

• VF quadrants; superonasal (SN), superotemporal (ST), inferonasal (IN), and inferotemporal (IT): Included as an exploratory segmentation to examine whether broader VF divisions reveal differences between GLA and HCs.

• VF halves; superior, inferior, temporal and nasal: The superior-inferior split reflect the typical asymmetry of glaucomatous damage across the horizontal meridian, whereas the nasal-temporal split was included exploratively to assess whether additional asymmetries could be captured.

• Absolute difference of opposing hemifields (superior-inferior, temporal-nasal), included to highlight intra-eye asymmetry.

• Mean and SD of all the VF points was calculated to highlight the overall loss of responsiveness and its variability within the field.

### Statistical analysis

All statistical analysis was performed separately for the best and worst eyes.

The power analysis:

The primary purpose of this study was to validate the Bulbicam VF test and identify biomarkers for use in GLA patients. In such studies, it is crucial to minimise false positive biomarker identification while avoiding the oversight of important biomarkers. Thus, the clinically relevant difference (CRD) between patients and HCs was set to 2 standard deviations (SD). With a significance level of 5% (α=0.05), and a power of 90% (β=0.90), a sample size of 12 patients and 12 HCs was required. Validity verification also includes documenting reliability and stability. For this purpose, a slightly larger sample size was considered appropriate. If the CRD was set to 1.5 SD with a corresponding significance level and power, the required number of patients and HCs increased to 16 in each.

*Validation:* The assumed continuously distributed variables were expressed as mean values with 95% confidence interval (CI). As an index of dispersion, SD or standard error (SE) were provided. All tests were performed two-tailed with a significance level of 5%. Analysis of Variance (ANOVA) and Receiver Operating Characteristic (ROC) analysis were used for group comparisons.

*Repeatability:* Let SD_w_ and SD_b_ denote the SD within and between participants, respectively, and M1 and M2 represent measurement 1 and 2. The Agreement Index (AI) derived from the Bland-Altman model, was used as a measure of repeatability within participants, defined as AI = 1 - $\frac{2SDw}{Mean\;of\;M1\;and\;M2}$ (33). Intraclass Correlation Coefficient version 3.1 (ICC) was used as measure of repeatability between participants. ICC values were calculated with the 2-way mixed effects absolute-agreement model (34), where $ICC=\;\frac{\underset{˙}{\text{O}}{'}_{b}^{2}}{\underset{˙}{\text{O}}{'}_{b}^{2}+\underset{˙}{\text{O}}{'}_{w}^{2}}$. This value represents the proportion of total variance in SRT measurements that is attributable to true differences between participants rather than measurement inconsistency within participants.

*Reliability:* A variable is considered reliable when both AI and ICC are ≥ 0.50, in conjunction with sufficient stability.

Stability: Stability was quantified as the Stability Index (SI), defined as SI = 1 - SDw/SDb, where SDw and SDb represent the SD within and between patients, respectively (35). The stability of a variable is considered acceptable when SI ≥ 0.14. Further details regarding the statistical approach can be found in the paper by Dalbro et al., 2025 (35).

*Biomarker:* A clinically useful biomarker must be valid and reliable. Validity is shown by group discrimination (ANOVA p<0.05 and/or ROC AUC with 95% CI lower bound >0.50). Reliability is defined by ICC (between participant repeatability), AI (within participant repeatability), and SI (temporal stability over several measurements). A variable is a population-level biomarker if validity criteria are met and ICC ≥ 0.50 and SI ≥ 0.14. An individual-level biomarker if validity criteria are met and AI ≥ 0.50 and SI ≥ 0.14. Variables meeting all four criteria (validity, AI, ICC and SI) qualify at both levels. All thresholds were pre-specified.

Statistical analyses were performed using SAS software version 9.4 (SAS Institute Inc., Cary, NC, USA).

## Results

### Visual field point analysis

**Figure 2:**
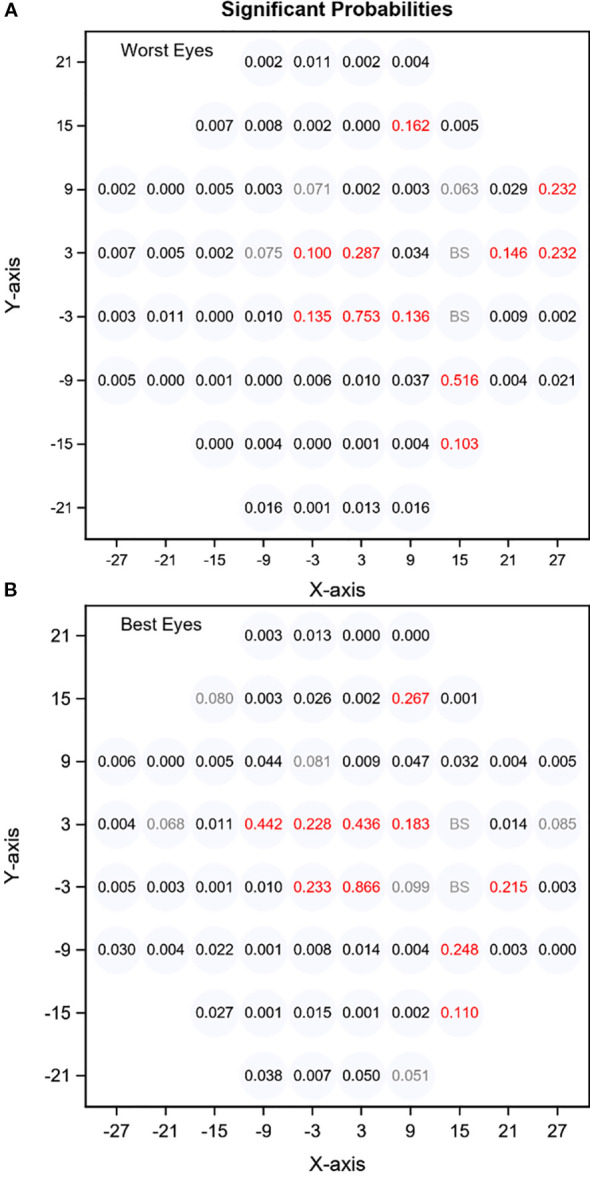
ANOVA probability plot of all VF points in the worst **(A)** and best **(B)** eyes.

In the worst eye, 44 out of 58 VF locations showed a significant difference compared to HC. No significant differences (p>0.05) were detected at 14 locations (**Figure 2A**). In the best eye, significant differences were observed in 42 locations, and 16 showed no significant difference (**Figure 2B**).

### Cluster analysis

**Figure 3:**
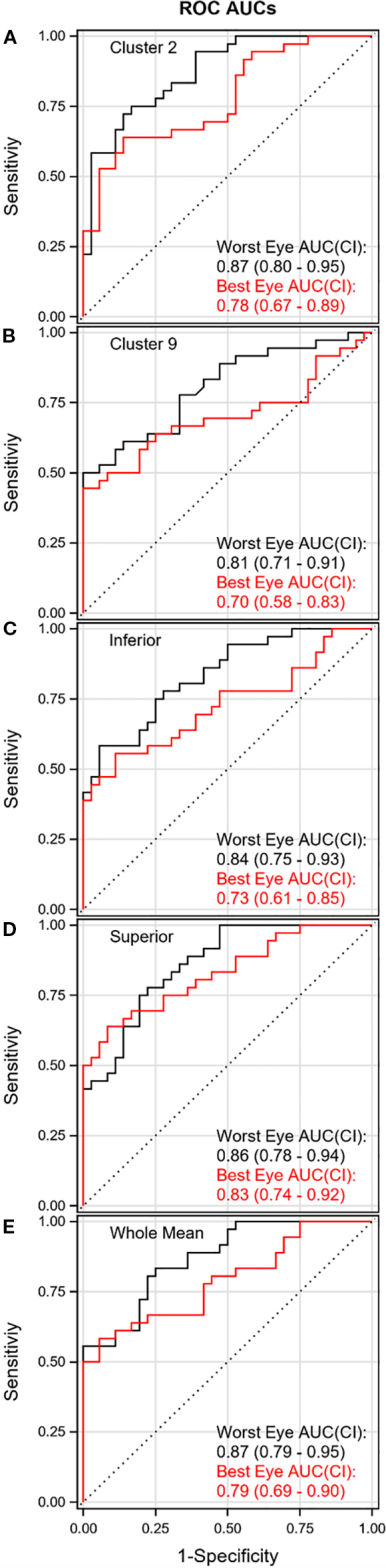
ROC curve for worst (black) and best (red) eyes.

SRT was significantly greater in GLA patients compared to HC in the ANOVA analysis, with significant differentiation confirmed by ROC analysis in 9 of the 10 VF clusters in the worst eye (**Table 2**, **Figures 3A, B**). For the best eye, significant differences between patients and HC were detected in 8 of the 10 clusters. ROC-analysis confirmed significant differentiation when the lower limit of the 95% confidence interval (CI) for AUC exceeded 0.50. No significant difference between patients and HC was detected in cluster 6 for either eye, or in cluster 5 for the best eye.

**Table 2:** Validation of the BCAM VF test. Comparison of patients with glaucoma and age-matched HCs including ROC analysis.

Eye	Saccadic reaction time (SRT)		GLA patients	Health controls	GLA-HC mean (95% CI)	ROC analysis	
						AUC	95% CI
Worst Eye	Cluster	1	772.6 (51.7)	498.6 (51.7)	274.0 (128.4-419.7)	0.78	0.67 - 0.89
		2	879.5 (46.6)	526.7 (46.6)	352.7 (221.3 - 484.1)	0.87	0.80 - 0.95
		3	799.9 (36.4)	553.2 (36.4)	246.7 (144.0 - 349.4)	0.81	0.71 - 0.91
		4	617.1 (41.2)	494.9 (41.2)	122.3 (6.0 - 238.5)	0.66	0.53 - 0.79
		5	749.4 (71.6)	531.1 (71.6)	218.3 (16.5 - 420.1)	0.64	0.51 - 0.77
		6	606.2 (47.3)	522.6 (47.3)	83.6 (-49.9 - 217.0)	0.58	0.45 - 0.72
		7	784.8 (47.2)	537.6 (47.2)	247.2 (114.0 - 380.5)	0.72	0.60 - 0.84
		8	828.3 (39.8)	588.9 (39.8)	239.4 (127.3 - 351.5)	0.76	0.65 - 0.87
		9	911.7 (49.6)	553.6 (49.6)	358.1 (218.2 - 498.0)	0.81	0.71 - 0.91
		10	869.6 (57.3)	502.8 (57.3)	366.8 (205.3 - 528.3)	0.84	0.75 - 0.93
	Quadrant	Superotemporal	760.1 (33.4)	521.9 (33.4)	238.3 (144.1 - 332.4)	0.82	0.73 - 0.91
		Inferotemporal	736.6 (29.8)	544.4 (29.8)	192.2 (108.0 - 276.4)	0.76	0.65 - 0.87
		Inferonasal	907.0 (52.9)	538.5 (52.9)	368.5 (219.3 - 517.6)	0.84	0.75 - 0.93
		Superonasal	834.9 (46.2)	528.3 (46.2)	306.6 (176.5 - 436.8)	0.84	0.75 - 0.93
	Half	Inferior	828.5 (37.2)	547.3 (37.2)	281.2 (176.2 - 386.2)	0.84	0.75 - 0.93
		Superior	794.9 (34.8)	529.9 (34.8)	265.0 (166.8 - 363.3)	0.86	0.78 - 0.94
		Abs diff/In-Su/	144.7 (16.4)	40.3 (16.4)	104.3 (58.0 - 150.6)	0.75	0.63 - 0.87
		Nasal	894.1 (48.2)	547.5 (48.2)	346.6 (210.7 - 482.5)	0.87	0.79 - 0.95
		Temporal	732.7 (26.4)	528.8 (26.4)	203.9 (129.5 - 278.4)	0.83	0.73 - 0.92
		Abs diff/Na-Te/	212.4 (27.7)	63.5 (27.7)	148.9 (70.8 - 227.0)	0.80	0.71 - 0.90
	Whole	Mean	811.5 (34.1)	538.5 (34.1)	273.0 (176.8 - 369.2)	0.87	0.79 - 0.95
		SD	491.8 (26.2)	243.6 (26.2)	248.2 (174.3 - 322.1)	0.87	0.79 - 0.95
Best Eye	Cluster	1	670.5 (46.1)	471.1 (46.1)	199.4 (69.4 - 329.4)	0.75	0.63 - 0.86
		2	741.3 (34.7)	499.9 (34.7)	241.4 (143.5 - 339.4)	0.78	0.67 - 0.89
		3	803.8 (41.0)	509.3 (41.0)	294.5 (178.9 - 410.1)	0.82	0.72 - 0.92
		4	624.3 (39.9)	434.7 (39.9)	189.6 (77.2 - 302.0)	0.67	0.54 - 0.80
		5	650.9 (48.4)	548.4 (48.4)	102.5 (-34.1 - 239.1)	0.64	0.51 - 0.76
		6	520.2 (25.1)	475.1 (25.1)	45.1 (-25.8 - 116.0)	0.53	0.39 - 0.66
		7	669.3 (43.3)	508.0 (43.3)	161.3 (39.3 - 283.3)	0.65	0.52 - 0.78
		8	783.4 (46.8)	587.1 (46.8)	196.4 (64.5 - 328.2)	0.64	0.51 - 0.77
		9	807.4 (48.4)	522.8 (48.4)	284.5 (148.0 - 421.0)	0.70	0.58 - 0.83
		10	713.3 (38.0)	468.7 (38.0)	244.6 (137.4 - 351.8)	0.77	0.66 - 0.88
	Quadrant	Superotemporal	746.4 (30.8)	510.5 (30.8)	235.9 (149.0 - 322.9)	0.84	0.76 - 0.93
		Inferotemporal	698.9 (34.2)	536.2 (34.2)	162.6 (66.1 - 259.2)	0.69	0.57 - 0.82
		Inferonasal	743.4 (37.2)	496.4 (37.2)	247.0 (142.2 - 351.8)	0.73	0.61 - 0.85
		Superonasal	703.5 (34.9)	485.4 (34.9)	218.0 (119.5 - 316.6)	0.79	0.68 - 0.89
	Half	Inferior	730.3 (33.7)	517.6 (33.7)	212.7 (117.6 - 307.8)	0.73	0.61 - 0.85
		Superior	724.9 (29.2)	499.2 (29.2)	225.7 (143.3 - 308.1)	0.83	0.74 - 0.92
		Abs diff/In-Su/	130.9 (14.5)	38.3 (14.5)	92.5 (51.6 - 133.4)	0.77	0.66 - 0.88
		Nasal	744.0 (35.4)	504.2 (35.4)	239.8 (139.8 - 339.7)	0.76	0.64 - 0.87
		Temporal	710.8 (27.3)	513.4 (27.3)	197.4 (120.5 - 274.3)	0.80	0.70 - 0.90
		Abs diff/Na-Te/	136.3 (15.4)	66.6 (15.4)	69.7 (26.4 - 113.1)	0.67	0.55 - 0.80
	Whole	Mean	727.8 (29.6)	508.5 (29.6)	219.2 (135.9 - 302.6)	0.79	0.69 - 0.90
		SD	430.5 (26.5)	215.9 (26.5)	214.6 (139.7 - 289.4)	0.80	0.70 - 0.91

The results are expressed by mean values, SE and 95% confidence intervals (CI).

**Figure 4:**
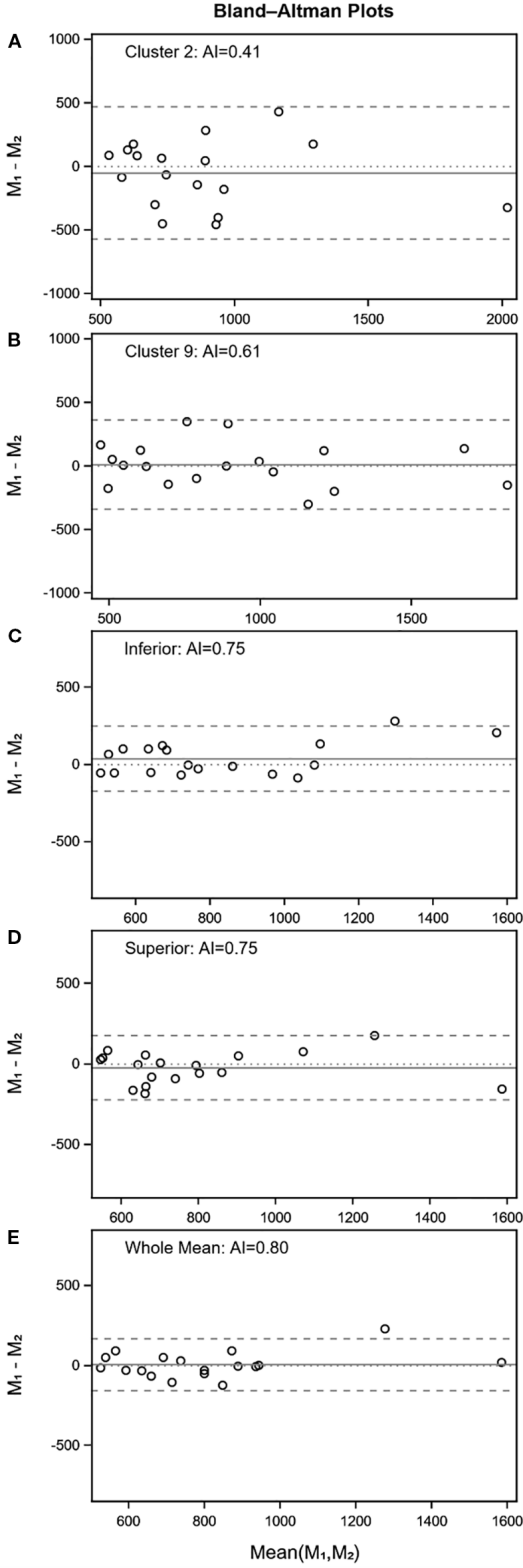
Bland-Altman plot with AI (worst eye only) for cluster 2 and 9, inferior and superior hemifield, and for the whole VF.

Between-patient repeatability (ICC ≥ 0.5) was not achieved for cluster 4 and 6 in the worst eye, nor for clusters 5 and 6 in the best eye (**Table 3**). However, the remaining clusters were found to be repeatable between patients. Within-patient repeatability was achieved for clusters 3 and 9 in the worst eye (**Figure 4B**), and for cluster 3 and 10 in the best eye.

**Table 3:** Reliability of the BCAM VF test expressed by ICC and AI.

Eye	Items	Variables	Glaucoma patients					Healthy controls				
			M1	M2	M1 – M2	ICC	AI	M1	M2	M1 – M2	ICC	AI
Worst Eye	Cluster	1	711.5	833.7	-122 (–403–159)	0.79	0.31	476.3	520.8	-45 (-142 - 53)	0.25	0.29
		2	854.0	904.9	-51 (-303-202)	0.76	0.41	528.8	524.7	4 (-95 - 103)	0.43	0.40
		3	808.2	791.5	17 (-159-192)	0.85	0.64	542.2	564.2	-22 (-142 - 98)	0.63	0.45
		4	618.5	615.7	3 (-187-192)	0.45	0.05	519.3	470.5	49 (-98 - 196)	0.30	-0.04
		5	836.6	662.3	174 (-212- 561)	0.56	-0.43	467.7	594.5	-127 (-265- 12)	-0.18	-0.18
		6	632.1	580.3	52 (-166- 270)	0.28	-0.27	461.9	583.4	-121 (-285- 42)	0.10	-0.24
		7	803.1	766.5	36.6 (-205-278)	0.70	0.30	544.2	530.9	13 (-119 - 146)	0.10	0.03
		8	817.6	839.0	-21 (-221 - 178)	0.57	0.34	593.6	584.2	9 (-109 - 128)	0.51	0.41
		9	917.4	905.9	11.5 (-261 - 284)	0.90	0.61	537.1	570.1	-33 (-130 - 64)	0.68	0.58
		10	920.5	818.7	102 (-221- 425)	0.88	0.46	498.8	506.7	-8 (-84 - 68)	0.74	0.68
	Quadrant	Superotemporal	742.8	777.5	-35 (-207- 137)	0.84	0.62	511.7	532.0	-20 (-110 - 70)	0.70	0.61
		Inferotemporal	731.4	741.7	-10(-160 - 139)	0.73	0.56	535.5	553.2	-18 (-106 - 71)	0.69	0.62
		Inferonasal	941.6	872.3	69 (-228- 366)	0.96	0.71	526.5	550.6	-24 (-103 - 55)	0.67	0.65
		Superonasal	829.4	840.3	-11 (-265- 243)	0.93	0.66	513.0	543.5	-30 (-118 - 58)	0.50	0.51
	Half	Inferior	847.4	809.7	38 (-164- 239)	0.94	0.75	535.2	559.5	-24 (-104 - 55)	0.74	0.69
		Superior	783.1	806.7	-24 (-209- 162)	0.93	0.75	519.4	540.3	-21 (-103 - 61)	0.80	0.71
		Abs diff/In-Su/	152.5	136.9	16 (-77-108)	0.80	-0.18	35.4	45.3	-10 (-34 - 14)	0.26	-1.17
		Nasal	910.0	878.2	31.9 (-236-299)	0.98	0.83	533.3	561.7	-28 (-113 - 57)	0.61	0.60
		Temporal	724.1	741.4	-17 (-146- 112)	0.82	0.69	520.7	536.8	-16 (-100 - 68)	0.81	0.71
		Abs diff/Na-Te/	215.2	209.6	6 (-149-160)	0.93	0.19	39.9	87.0	-47 (-90 - -4)	-0.14	-2.02
	Whole	Mean	814.6	808.4	6 (-176-189)	0.95	0.80	527.3	549.8	-23 (-101 - 56)	0.80	0.73
		SD	484.0	499.2	-15 (-147-118)	0.91	0.67	193.9	293.3	-99 (-167- -32)	0.62	0.29
Best Eye	Cluster	1	673.3	667.8	6 (-249-260)	0.70	0.31	430.3	512.0	-82 (-164 - 0)	0.14	0.33
		2	724.4	758.2	-34 (-217-149)	0.72	0.45	520.4	479.3	41 (-44 -127)	0.47	0.48
		3	781.9	825.6	-45 (-269-182)	0.86	0.56	485.8	532.7	-47 (-123 - 29)	0.39	0.51
		4	669.0	579.7	89 (-125- 303)	0.55	0.04	449.1	420.4	29 (-56 - 113)	0.03	0.20
		5	662.1	639.6	23 (-199- 244)	0.09	-0.35	511.2	585.6	-74 (-247 - 98)	-0.07	-0.36
		6	534.4	506.0	28 (-102-159)	0.39	0.18	471.7	478.5	-7 (-72 - 59)	0.21	0.49
		7	638.3	700.4	-62 (-293-169)	0.82	0.38	503.8	512.3	-9 (-109 - 91)	0.48	0.41
		8	734.0	832.9	-99 (-349-151)	0.72	0.29	583.9	590.2	-6 (-109 - 96)	0.61	0.55
		9	817.0	797.7	19 (-255- 293)	0.80	0.36	509.7	536.0	-26 (-93 - 41)	0.57	0.65
		10	741.5	685.0	57 (-154- 267)	0.90	0.61	454.5	482.9	-28 (-93 - 36)	0.59	0.63
	Quadrant	Superotemporal	740.3	752.6	-12 (-172- 148)	0.78	0.58	504.3	516.7	-12 (-94 - 69)	0.35	0.46
		Inferotemporal	688.5	709.2	-21 (-203-162)	0.77	0.48	529.4	543.0	-14 (-95 - 67)	0.65	0.63
		Inferonasal	749.6	737.1	13 (-196- 221)	0.91	0.65	485.6	507.2	-22 (-78 - 35)	0.42	0.64
		Superonasal	698.1	708.8	-11 (-204-183)	0.82	0.51	467.7	503.2	-35 (-99 - 28)	0.46	0.60
	Half	Inferior	733.8	726.7	7 (-180-194)	0.95	0.76	509.5	525.7	-16 (-76 - 43)	0.62	0.70
		Superior	726.7	723.1	4 (-157- 164)	0.92	0.74	488.5	510.0	-22 (-80 - 36)	0.64	0.71
		Abs(In-Su)	137.8	123.9	14 (-68- 95)	0.54	-0.77	48.6	28.0	21 (0 - 42)	0.10	-1.17
		Nasal	752.0	735.9	16 (-182- 214)	0.96	0.77	491.2	517.1	-26 (-86 - 34)	0.50	0.65
		Temporal	706.8	714.7	-8 (-152- 136)	0.83	0.65	507.8	518.9	-11 (-79 - 57)	0.64	0.67
		Abs (Na-Te)	167.2	105.5	62 (-17- 141)	0.43	-0.83	47.7	85.5	-38 (-72 - -4)	-0.06	-1.20
	Whole	Mean	729.9	725.6	4 (-158-167)	0.96	0.82	499.2	517.9	-19 (-76 - 38)	0.72	0.75
		SD	437.2	423.7	14 (-124 – 151)	0.86	0.50	195.2	236.6	-41 (-111 - 28)	0.24	-0.17

The results are expressed by Least square Mean (LSM) value, SE and 95% CI.

**Figure 5:**
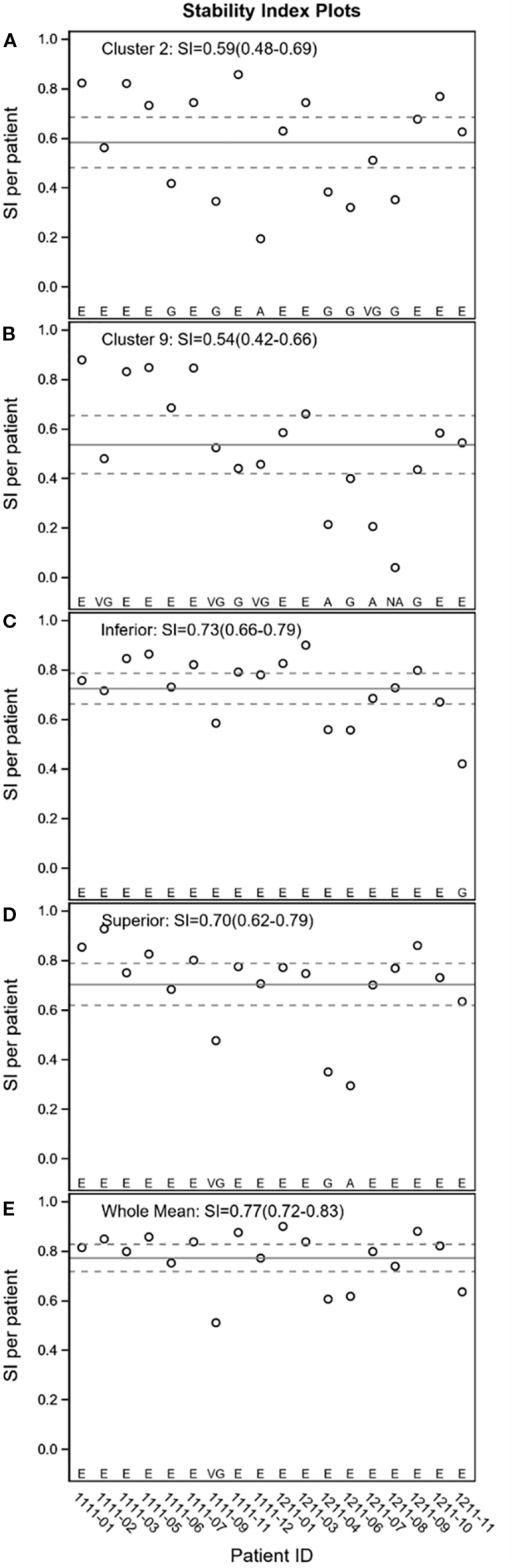
Stability Index (worst eye only) for cluster 2 and 9, inferior and superior hemifield, and for the whole VF.

The stability of SRT was sufficient across all 10 clusters for both the worst and the best eye (**Table 4**). In the worst eye, stability was classified as “Excellent” in 4 clusters, “Very Good” in 2 clusters, and “Good” in 4 clusters (**Figures 5A, B**). Similarly, for the best eye, 3 clusters were classified as “Excellent”, 2 as “Very Good” and 5 as “Good”.

**Table 4:** Stability of the BCAM VF test.

Eye	Variable		M1	M2	M3	M4	M5	M6	SI (95%CI)	Cla
Worst Eye	Cluster	1	711.5	833.7	896.2	795.3	813.1	814.1	0.50 (0.31-0.69)	VG
		2	854.0	904.9	856.0	847.4	840.0	822.2	0.59 (0.48-0.69)	E
		3	808.2	791.5	739.0	863.3	801.1	864.6	0.43 (0.21-0.65)	G
		4	618.5	615.7	643.8	639.3	617.3	608.2	0.39 (0.23-0.54)	G
		5	836.6	662.3	784.0	693.4	602.5	573.6	0.60 (0.38-0.82)	E
		6	632.1	580.3	571.2	617.4	531.1	588.7	0.52 (0.32-0.73)	VG
		7	803.1	766.5	714.7	722.8	695.2	758.6	0.40 (0.23-0.58)	G
		8	817.6	839.0	840.7	858.1	829.4	842.4	0.35 (0.18-0.53)	G
		9	917.4	905.9	976.1	885.7	892.1	955.3	0.54 (0.42-0.66)	E
		10	920.5	818.7	841.7	910.8	910.9	904.7	0.76 (0.69-0.83)	E
	Quadrate	Superotemporal	742.8	777.5	727.2	756.7	764.9	738.5	0.54 (0.43-0.65)	E
		Inferotemporal	731.4	741.7	735.5	763.9	750.0	745.6	0.43 (0.24-0.62)	G
		Inferonasal	941.6	872.3	894.6	911.9	886.7	941.2	0.75 (0.69-0.81)	E
		Superonasal	829.4	840.3	864.7	848.8	814.8	831.8	0.69 (0.59-0.79)	E
	Half	Inferior	847.4	809.7	828.4	846.6	826.0	847.1	0.73 (0.66-0.79)	E
		Superior	783.1	806.7	807.9	806.0	793.4	782.2	0.70 (0.62-0.79)	E
		Abs diff/In-Su/	152.5	136.9	129.9	134.7	165.5	169.4	0.49 (0.36-0.63)	VG
		Nasal	910.0	878.2	910.6	907.1	874.5	901.5	0.79 (0.74-0.85)	E
		Temporal	724.1	741.4	728.3	746.6	751.0	727.5	0.57 (0.48-0.67)	E
		Abs diff/Na-Te/	215.2	209.6	246.1	285.3	224.1	261.2	0.58 (0.47-0.70)	E
	Whole	Mean	814.6	808.4	818.6	825.9	810.0	814.2	0.77 (0.72-0.83)	E
		SD	484.3	499.2	481.4	516.0	499.7	521.9	0.66 (0.61-0.72)	E
Best Eye	Cluster	1	673.3	667.8	701.1	683.6	670.8	703.5	0.65 (0.51-0.79)	E
		2	724.4	758.2	710.9	705.4	664.3	743.3	0.54 (0.42-0.66)	E
		3	781.9	825.6	777.2	901.8	781.6	847.9	0.46 (0.25-0.67)	VG
		4	669.0	579.7	652.9	654.8	609.4	566.0	0.46 (0.28-0.64)	VG
		5	662.1	639.6	619.1	655.7	608.7	549.5	0.39 (0.14-0.64)	G
		6	534.4	506.0	471.2	537.4	546.4	548.5	0.35 (0.01-0.68)	G
		7	638.3	700.4	659.6	697.6	736.6	583.9	0.41 (0.14-0.67)	G
		8	734.0	832.9	848.7	847.8	809.0	833.3	0.39 (0.24-0.54)	G
		9	817.0	797.7	895.0	739.5	868.7	1011.9	0.44 (0.25-0.64)	G
		10	741.5	685.0	654.4	731.3	682.8	681.0	0.69 (0.61-0.77)	E
	Quadrate	Superotemporal	740.3	752.6	714.3	774.4	747.7	741.6	0.34 (0.18-0.51)	G
		Inferotemporal	688.5	709.2	737.5	709.1	741.7	717.7	0.50 (0.38-0.62)	VG
		Inferonasal	749.6	737.1	717.0	755.3	740.1	784.1	0.67 (0.59-0.76)	E
		Superonasal	698.1	708.8	704.6	709.1	639.6	717.6	0.70 (0.59-0.80)	E
	Half	Inferior	733.8	726.7	731.3	741.5	745.9	759.2	0.70 (0.63-0.77)	E
		Superior	726.7	723.1	716.1	740.9	704.4	730.7	0.68 (0.58-0.77)	E
		Abs diff/In-Su/	137.8	123.9	122.2	123.9	137.9	143.0	0.48 (0.33-0.63)	VG
		Nasal	752.0	735.9	728.8	750.0	712.1	770.5	0.75 (0.68-0.82)	E
		Temporal	706.8	714.7	718.5	730.7	739.2	716.0	0.50 (0.38-0.62)	VG
		Abs diff/Na-Te/	167.2	105.5	142.4	178.8	153.9	129.4	0.41 (0.25-0.56)	G
	Whole	Mean	729.9	725.6	723.8	741.1	726.4	743.9	0.72 (0.65-0.80)	E
		SD	437.2	423.7	430.8	421.1	436.6	450.5	0.59 (0.47-0.70)	E

The six measurements are denoted as M1 to M6 and expressed by mean values. Stability is expressed by the Stability Index (SI) with 95% Confidence Intervals (CI). The classification is based on SI. Classification (Cla), E, Excellent; VG, Very Good; G, Good.

In the worst eye, clusters 3 and 9 qualify as BCAM biomarkers for GLA (**Table 5**). Clusters 1, 2, 5, 7, 8 and 10 are potential biomarkers, but require re-testing on the same patient, as within-participant repeatability was classified as “poor”, with sufficient stability. Clusters 4 and 6 are not recommended as BCAM biomarkers for GLA.

**Table 5:** Recommended user area as Bulbicam Visual Field Biomarkers for Glaucoma.

Possible biomarker for glaucoma		ROC	ICC	AI	SI	Recommended user area	
						Worst eye	Best eye
Cluster	1	+	+	–	+	Population + Patient*^)^	Population + Patient*^)^
	2	+	+	–	+	Population + Patient*^)^	Population + Patient*^)^
	3	+	+	+	+	Population + Patient	Population + Patient
	4	+	–	–	+	Not recommended	Population + Patient*^)^
	5	+	+	–	+	Population + Patient*)	Not recommended
	6	–	–	–	+	Not recommended	Not recommended
	7	+	+	–	+	Population + Patient*)	Population + Patient*)
	8	+	+	–	+	Population + Patient*^)^	Population + Patient*^)^
	9	+	+	+	+	Population + Patient	Population + Patient*^)^
	10	+	+	–	+	Population + Patient*^)^	Population + Patient
Quadrant	Superotemporal	+	+	+	+	Population + Patient	Population + Patient
	Inferotemporal	+	+	+	+	Population + Patient	Population + Patient*)
	Inferonasal	+	+	+	+	Population + Patient	Population + Patient
	Superonasal	+	+	+	+	Population + Patient	Population + Patient
Half	Inferior	+	+	+	+	Population + Patient	Population + Patient
	Superior	+	+	+	+	Population + Patient	Population + Patient
	Abs diff/In-Su/	+	+	–	+	Population + Patient*^)^	Population + Patient*^)^
	Nasal	+	+	+	+	Population + Patient	Population + Patient
	Temporal	+	+	+	+	Population + Patient	Population + Patient
	Abs diff/Na-Te/	+	+	–	+	Population + Patient*^)^	Not recommended
Whole	Mean	+	+	+	+	Population + Patient	Population + Patient
	SD	+	+	+	+	Population + Patient	Population + Patient

*^)^ Need to be repeated on patient level. ROC, ICC, AI, and SI notations are only shown for worst eye.

### Quadrant analysis

SRT was significantly greater among patients compared to HC for all VF quadrants in both the worst and the best eyes (**Table 2**). SRT was found to be repeatable between and within patients for all quadrants.

Both the IN and the SN quadrants demonstrated high reliability with excellent repeatability (**Table 3**) and stability (**Table 4**). Stability classifications were “Excellent” for both quadrants in both eyes. ST and IT quadrants showed “Excellent” and “Very Good” stability in the worst eye, and “Good” and “Excellent” stability in the best eye, respectively.

With the exception of the IT quadrant in the best eye, which is classified as a potential biomarker for GLA due to an AI below 0.5, all VF quadrants are classified as BCAM biomarkers for GLA in both eyes.

### Hemifield analysis

The SRT in all hemifields, as well as the absolute difference between opposing hemifields, was significantly greater in GLA patients compared to HC for both eyes (**Table 2**, **Figures 3C, D**).

All hemifields were found to be repeatable between patients, with the exception of the absolute difference between the temporal and nasal hemifields (**Table 3**). Within-patient repeatability was satisfactory for all hemifields in both eyes, except for absolute differences (**Figures 4C, D**).

Stability was found to be sufficient in all hemifields for both eyes (**Table 4**). In the worst eye, stability was classified as “Excellent” for all hemifields, except for the absolute difference between the superior and inferior hemifields, which was classified as “Very Good” (**Figures 5C, D**).

SRT for all hemifields qualifies as BCAM biomarkers for GLA. However, the absolute difference between opposing hemifields has potential as a biomarkers but requires re-testing on the same patient, as the repeatability was “poor” (AI<0.5), with stability classified as “Very Good”.

### Whole-area analysis

The mean and SD of SRTs across the entire VF were significantly greater in GLA patients compared to HC for both eyes (**Table 1**). AUC values in ROC analysis exceeded 0.5 with high confidence for both eyes (**Figure 3E**).

Both variables demonstrated repeatability between- and within-patients for both eyes (**Table 3**, **Figure 4E**); however, within-patient repeatability for SD in the best eye was borderline.

Stability was found to be sufficient for both variables in both eyes (**Table 4**) and was classified as “Excellent” (**Figure 5E**). Both the mean and SD of SRTs qualify as BCAM biomarkers for GLA in both eyes.

## Discussion

This study demonstrates that multiple SRT-based variables measured with the BCAM system are capable of differentiating GLA from HCs, with sufficient reliability, especially when VF points were aggregated into larger regions. These findings support their potential as biomarkers for GLA. Moreover, our results extend prior work by systematically evaluating both validity and reliability of SRT-based EMP in a GLA population, an important step for clinical translation.

Prolonged SRT in GLA has been well-documented in the literature, and our findings align with this body of evidence. Mean SRT in our GLA group were 43% (best eye) and 50.7% (worst eye) longer than in HCs, consistent with prior studies reporting increases ranging from 7.2-54% depending on methodology and disease stage (14–16, 27). The strong discriminatory performance of global mean SRT, even in mild to moderate GLA suggests that SRT may be particularly sensitive to glaucomatous damage. This is in line with previous work showing SRT to be prolonged even in regions with normal light sensitivity (25–27). Elgin also reported prolonged SRTs in preperimetric and moderate GLA, particularly using a kinetic paradigm, suggesting its increased sensitivity to glaucomatous damage. Moreover, by applying a machine learning approach to patterns across multiple SEM variables, the study achieved an AUC of 0.87 for detecting preperimetric GLA (27).

In our study, most VF locations showed significant differentiation between GLA and HCs. Using a similar grid, Meethal et al. (36) found slightly higher mean pointwise AUC of 0.75 (0.05) compared with our findings of 0.67 (0.06) and 0.7 (0.06) for the best and worst eye respectively. This likely reflects their inclusion of advanced glaucoma together with differences in stimulus settings.

Test-retest variability is a well-known limitation of SAP in GLA, where results often fluctuate more than in healthy individuals (11, 37). Pel et al. (28) found low variability of SRT across three measurement series in healthy subjects. In our study, test-retest variability was comparable between GLA and HCs, as reflected by the similar AI classifications. Only 3 of the 22 variables differed between groups, suggesting that, under our protocol, SRT-based measures are not disproportionately susceptible to disease-related variability, consistent with findings from frequency doubling- and motion perimetry (38–40).

Nonetheless, some test-retest variability was present, which may partly be related to stimulus characteristics and fluctuation in fatigue and attention. The overlap paradigm was selected to reduce express saccades, the trade-off may be wider SRT distributions (13, 41, 42). To promote reflexive saccades, flickering and pseudorandom stimuli were applied to enhance salience, yet a proportion of voluntary or predictive saccades likely contributed additional variability (43).

Aggregating single VF locations into larger areas improved the validity and reliability markedly. Although this is an expected consequence of averaging across multiple locations, it still represents a practical way of obtaining more reliable measures, particularly when clusters are organised to reflect the anatomical layout of the RNFL bundles (44).

In our study, only cluster 3 and 9 in the worst eye, and cluster 3 and 10 in the best eye, were reliable and stable. These clusters correspond to regions commonly affected in early-to-moderate GLA, including the superior arcuate and nasal/inferior paracentral VF (45, 46). The lack of reliability in other clusters likely relates to the relatively few VF points in each cluster and the heterogeneous impact of GLA on the VF responsiveness in the study sample. Aggregating points into VF quadrants and hemifields further improved the validity and reliability, with the nasal regions performing best, consistent with the notion of early glaucomatous damage often affecting on the nasal side of the VF (47–49).

Asymmetrical defects between the superior and inferior hemifields are another hallmark of GLA, related to asymmetric damage of the neuroretinal rim (48). Algorithms such as the glaucoma hemifield test (GHT) in the Humphrey Field Analyzer (HFA) use this principle to help detect early glaucomatous changes. We included a simple variable to highlight such asymmetry - the absolute difference between opposing hemifields. Contrary to our initial expectation, this variable underperformed relative to analysing each hemifield independently. This may reflect relatively symmetric field loss in our study sample, or a global SRT depression in GLA, as suggested previously (15, 25). Mazumdar et al. (15) reported an AUC of 0.78 using a sector-based approach analogous to the GHT. The simpler approach used in our study produced comparable values.

Beyond hemifield asymmetry, early GLA VF loss is often localised and heterogenous. Variability is typically captured by indices such as the square root of loss variance (Octopus) and pattern standard deviation (HFA). Using the SD of the entire VF plot, we found significantly greater variability in GLA patients compared to HC, with ROC-AUC values of 0.87 for the worst eye and 0.8 for the best eye.

Mean SRT across the VF was the most reliable variable, and produced the strongest discriminatory ability with AUC of 0.79 and 0.87 in the best and worst eye respectively. However, its specificity is limited, as SRT is affected by a range of other diseases (50). For this reason, a function-structure specific approach, such as cluster analysis, are likely to provide a more GLA specific evaluation.

From a practical standpoint, BCAM VF test completed a 24–2 pattern in roughly 2 to 2.5 minutes per eye, comparable to faster SAP strategies (51, 52), and integrates display and eye-tracking in a single unit, simplifying setup relative to earlier EMP systems. These features may facilitate clinical use if diagnostic performance is confirmed in broader cohorts.

By engaging natural oculomotor reflexes and increasing retinal image change, SRT-based perimetry may enhance engagement and reduce factors known to compromise reliability in SAP, including inattention, fixation loss, false positive and negative responses, the Troxler fading effect, and Ganzfeld blank-out (53–58).

Our study has several limitations that should be acknowledged. First, the study primarily focused on group-level differences between otherwise healthy GLA patients and controls. While these findings are promising, further research is needed to evaluate the performance of the BCAM VF test across a wider spectrum of patient profiles, including those with comorbidities and a broader range of disease severity. Second, glaucoma diagnoses were based on routine clinical judgement without prespecified case definition, introducing risk of misclassification. Third, the study design limits our ability to assess the performance to detect progression over time, which is critical for monitoring the slow nature of GLA. Fourth, all GLA participants were using at least one form of topical anti-glaucoma medication at the time of testing, and the potential influence of such medications on SRT remains unexplored, however, none reported using systemic medications known to affect SRT (59–62). Fifth, the study did not include a direct comparison with SAP, the current clinical benchmark in functional testing in GLA, which will be important in future evaluations. Finally, participants´ eyes were categorized as “worst” and “best”. This approach allowed for independent analysis of each eye, avoiding the need for complex statistical models, preventing the rejection of useful data, and reducing the required number of participants (63, 64). However, this approach also introduced greater variability in disease progression within the two groups, possibly obscuring clear trends.

## Conclusion

The findings demonstrate that the majority of the SRT variables studied are not only effective in differentiating glaucomatous eyes from HC, but also exhibit a sufficient level of reliability and stability, which is essential for use in a clinical setting. Furthermore, 19 of the 22 BCAM VF test variables were identified as potential GLA-biomarkers according to pre-specified criteria.

## Data Availability

The datasets presented in this article are not readily available because the raw datasets generated for this study contain sensitive patient information and are subject to strict data privacy regulations, including those derived from the General Data Protection Regulation (GDPR) and relevant Norwegian legislation (e.g., Personopplysningsloven). Consequently, direct public sharing of the full dataset is not possible due to patient confidentiality. The data is stored securely at Meddoc AS, who also performed the analysis. While the raw data cannot be openly shared, reasonable requests for access to de-identified aggregate data or for verification of key findings will be considered by the corresponding author upon approval from the data custodians and in accordance with applicable data protection laws. Requests to access the datasets should be directed to sl@meddoc.no.
